# A Simple and Safe Anastomosis for Pancreatogastrostomy Using One Binding Purse-String and Two Transfixing Mattress Sutures

**DOI:** 10.1155/2012/718637

**Published:** 2012-02-27

**Authors:** D. K. Bartsch, P. Langer, V. Kanngießer, V. Fendrich, K. Dietzel

**Affiliations:** Department of Surgery, Philipps University of Marburg, Baldingerstrasse 35043 Marburg, Germany

## Abstract

Pancreatic anastomotic leakage remains a persistent problem after pancreaticoduodenectomy (PD), especially in the presence of a soft, nonfibrotic pancreas. A modified technique for pancreatogastrostomy was devised, which combines one binding purse-string and two transfixing mattress sutures between the pancreatic stump and the posterior gastric wall. This technique was applied in 35 patients after PD for malignant and benign diseases of whom 10 (28.6%) had a soft pancreas. Median time for the anastomosis was 18 minutes. Operative mortality was zero, and morbidity was 34.3%. Three (8.6%) patients developed a pancreatic fistula (2 type A, 1 type B) as classified according to the International Study Group on pancreatic fistula. All fistulas resolved without further intervention. The described technique is a simple and safe reconstruction procedure after PD that warrants further evaluation.

## 1. Introduction

Pancreatic leakage remains a common and serious complication after standard pancreaticoduodenectomy (PD) or pylorus-preserving pancreaticoduodenectomy (PPPD) [1–3]. Pancreatic fistula is occasionally followed by several other potentially life-threatening complications, such as massive haemorrhage of eroded vessels and peritonitis. To prevent these complications, two main anastomotic techniques for reconstruction after PD, pancreaticojejunostomy (PJ), and pancreaticogastrostomy (PG), exist. According to four randomized trials comparing PJ and PG, there is no difference regarding the prevalence of pancreatic fistula between these reconstruction techniques [4–7]. However, it has been suggested that PG is associated with fewer overall complications than PJ [6, 8]. Several different methods of anastomosing the pancreas to the stomach have been employed, including PG using several mattress sutures [9] and the so-called binding PG using two purse-string sutures at the posterior gastric wall [10]. Here we report the early results of a new technique for PG, which combines one binding purse-string and two transfixing mattress sutures between the pancreatic stump and the posterior gastric wall. This technique is now used as a standard technique for reconstruction after PD.

## 2. Patients and Methods

The study cohort includes 35 patients who underwent elective PD with PG at the Department of Visceral, Thoracic and Vascular Surgery, Philipps University of Marburg, between June 2007 and February 2011. Patients data, including patients demographics (age, sex, diagnosis), type of procedure performed (standard PD or PPPD), complications, hospital mortality, hospital stay, postoperative interventional procedures or reoperations were prospectively documented. 

All patients had two closed suction drains placed at the time of operation in close proximity to the pancreatic anastomosis. Patients received octreotide (3 times 100 *μ*g/d) until postoperative day 5. The proton pump inhibitor omeprazole was given in a dosage two times 40 mg/day for 7 days. The nasogastric tube was left in place until postoperative day 5 to protect the pancreatogastrostomy. However, the patients were allowed to drink fluids as well as liquid meals at postoperative day 1. The closed suction drains were removed on postoperative day 5, when amylase was not more than three times higher than the serum amylase. The volume and amylase activity of the drainage fluid were measured on postoperative days 1, 3, 5 and thereafter, when a pancreatic fistula was present. Pancreatic fistula was classified according to the strict criteria of the IGSPF [11]. In brief, fistulas without clinical impact were therefore graded type A. Fistulas with maintenance of the drains longer than 3 weeks postoperatively were graded type B, whereas a type C fistula leads to clinical interventions like reoperations and percutaneous drainage. Delayed gastric empting (DGE) was defined as intolerance of an unlimited intake of any diet after postoperative day 7. Pancreatic texture was classified as soft or hard based on intraoperative findings and pathological examination.

## 3. Technique

**Figure 1:**
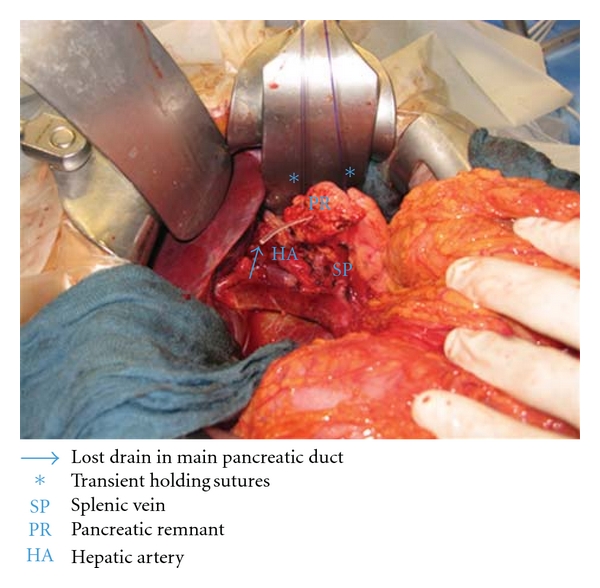
Mobilised pancreatic remnant with a lost drain inserted into the main pancreatic duct.

**Figure 2:**
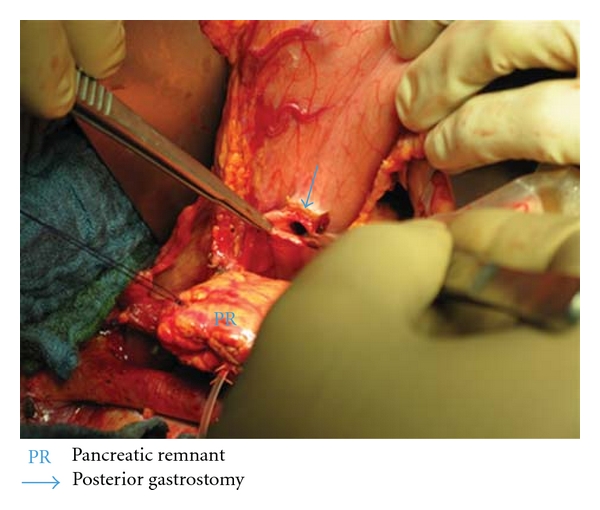
Position of small transverse gastrostomy on the posterior gastric wall.

**Figure 3:**
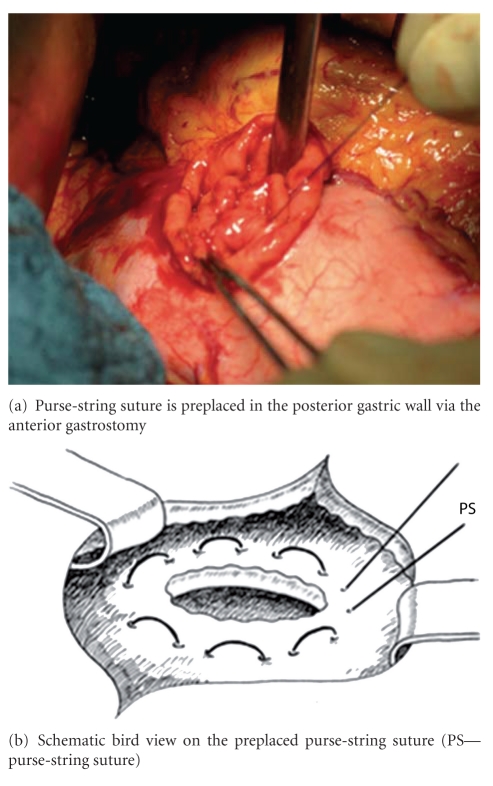
Preplaced purse-string suture (PDS II 2.0 MH plus, 70 cm) at the posterior gastric wall.

**Figure 4:**
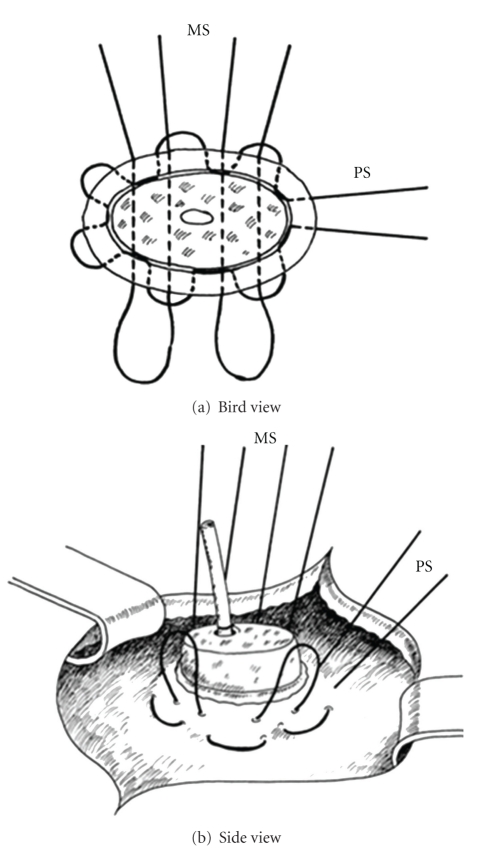
Scheme of the 2 mattress sutures running through the posterior gastric wall and the pancreatic remnant in an U-like fashion, while the purse-string suture is already in place. PS: purse-string suture; MS: mattress sutures.

**Figure 5:**
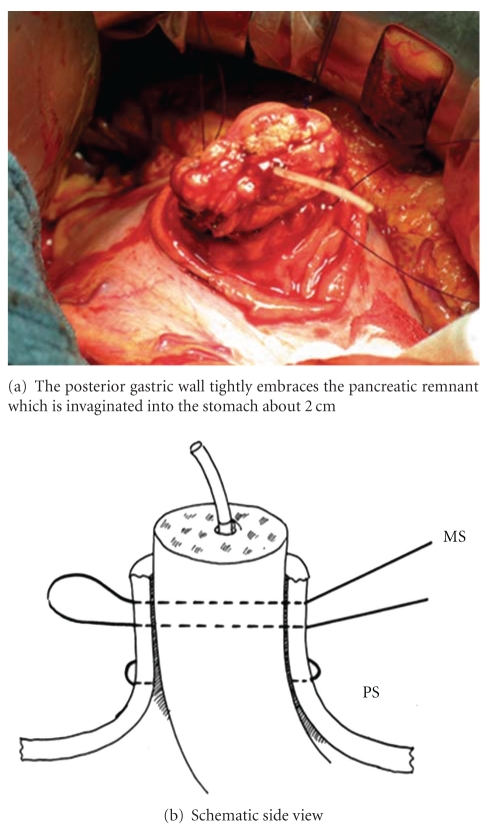
Situs after placement of the 2 mattress sutures and the purse-string suture (side view).

After the pancreaticoduodenectomy, any bleedings from the cut surface of the pancreatic stump were stopped by bipolar electrical coagulation or absorbable sutures (PDS 5.0, Ethicon, Johnson & Johnson Medical GmbH, Norderstedt, Germany). Then the pancreatic remnant was mobilized 2 to 3 cm from the splenic vein and the surrounding tissues. A polyethylene 5 cm pancreatic tube, 5.0 or 7.5 French (Peter Pflugbeil GmbH, Zorneding, Germany) was introduced into the main pancreatic duct to insure its patency. This lost drain was fixed to the main pancreatic duct by a 5.0 absorbable suture (vicryl rapide 5-0 P-3, 45 cm; Ethicon, Johnson & Johnson Medical GmbH, Norderstedt, Germany). Two transient holding sutures (vicryl plus 2.0 MH, 70 cm; Ethicon, Johnson & Johnson Medical GmbH, Norderstedt, Germany) were positioned at the cranial and caudal proximal end of the pancreatic remnant (Figure 1). Then a transverse full-thickness incision was made on the posterior wall of the stomach with a length of at most 2 cm, to ensure tight adherence of the gastric wall to the pancreatic remnant after completion of anastomosis (Figure 2). The appropriate position of the incision was selected, so that the pancreatic stump could enter this hole without tension. Then a 5-cm longitudinal incision was created in the anterior gastric wall opposite to the dorsal wall incision. Through the incision of the anterior gastric wall, a full-thickness purse-string suture (PDS II 2.0 MH plus, 70 cm; Ethicon, Johnson & Johnson Medical GmbH, Norderstedt, Germany), taking about 1 cm of the whole posterior gastric wall, was preplaced (Figures 3(a) and 3(b)). The pancreatic remnant was then pulled with slide tension on the holding sutures through the whole in the posterior gastric wall into the stomach. This manoeuvre was performed very gently to ensure tight wrapping of the posterior gastric wall around the pancreatic remnant and to avoid laceration of the pancreas. Ideally, the pancreatic remnant should protrude above the posterior gastric wall by 2 cm. Afterwards mattress sutures were preplaced through the posterior gastric wall and the pancreas, one cranial and one caudal of the main pancreatic duct. These sutures were carried out with double-armed straight needles (PDS II 4.0 MH, 70 cm; Ethicon, Johnson & Johnson Medical GmbH, Norderstedt, Germany) passing in an U-like fashion. Each U-like suture runs from the mucosal surface to the serosal surface of the caudal posterior gastric wall, just above the preplaced purse-string suture, then straight through the ventral to the dorsal surface of the pancreas, and finally from the serosal surface to the mucosal surface of the cranial posterior gastric wall (Figure 4). The threads near the centre of the pancreatic stump were placed carefully to avoid passing through the main pancreatic duct containing the catheter. First the U-like mattress sutures and then the purse-string suture were ligated (Figure 5). Finally, the pancreatic remnant was revised for any minor bleedings. A nasogastric tube was positioned just above the PG before closure of the anterior gastric wall. The gastrostomy on the anterior gastric wall was closed with all layer single sutures (PDS II 2-0 JB, 70 cm; Ethicon, Johnson & Johnson Medical GmbH, Norderstedt, Germany). An end-to-side hepaticojejunostomy and antecolic end-to-side gastrojejunostomy in case of standard PD or antecolic duodenojejunostomy in case of PPPD were performed to complete the reconstruction.

## 4. Results

### 4.1. Patients Characteristics

A total of 35 patients underwent PD with the new modified PG technique. There were 21 men and 14 women with a median age of 61.9 (range 25–84) years. Thirty (85.7%) patients underwent PPPD and 5 (14.3%) patients had standard PD. In 5 (14.3%) patients also a partial portal vein resection and reconstruction was performed. The indications for PD were as follows: 24 pancreatic adenocarcinomas, 6 neuroendocrine tumors of the pancreatic head or duodenum, 1 cancer of the papilla Vateri, 2 distal bile duct cancers, one IPMN and one chronic pancreatitis. Twenty-five patients (71.4%) had a firm pancreatic remnant, and 10 (28.6%) patients had a soft pancreatic remnant. The median time for conduction of the PG (incision of the posterior gastric wall to finishing the closure of the anterior gastric wall) was 18 (range 12–28) minutes. 

### 4.2. Complications

There were no operative or hospital deaths. Complications occurred in 12 (34.3%) patients (Table 1). The most frequent complication was DGE, which occurred in 7 (20%) patients. Pancreatic fistulas occurred in 3 (8.6%) patients (2x type A; 1x type B). The type A fistulas occurred in patients with a firm pancreas, the type B fistula in a patient with MEN1-ZES and a soft pancreas. Thus, a clinically relevant pancreatic fistula occurred in 1 (2.9%) of all or in 1 (10%) of 10 patients with a soft pancreatic remnant. All three pancreatic fistulas could be managed non-operatively by maintaining the closed suction drains (14, 15 and 30 days).

## 5. Discussion

Pancreaticogastrostomy (PG) is the favoured reconstruction procedure of several surgeons after pancreaticoduodenectomy, although 4 prospective randomised trials showed no difference regarding pancreatic fistula or overall complication rates compared to pancreaticojejunostomy (PJ) [4–6]. However, PG has some theoretical and technical advantages over PJ. First, the anastomosis can be created easily because of the proximity of the stomach and the pancreas remnant. Second, the thick posterior gastric wall is an excellent suture bed with an excellent blood supply. Third, the pancreatic juice is not activated because of the acid milieu and lack of enterokinases in the stomach. Fourth, if a minor dehiscence of the PG occurs, it can be managed in many cases via endoscopic interventions, for instance, fibrin sealing. 

Since the first clinical application of PG performed by Waugh and Clagett in 1946 [12], a large variety of modifications of the technique has been published in the literature (Table 2). These modifications include invagination or duct-to-mucosa anastomosis, use of transanastomotic tubes for internal-external drainage of pancreatic juice, use of fibrin sealant, use of multiple transfixing mattress sutures [9] or 2 purse-string binding sutures [10], respectively. Recently published studies on PG had shown that the prevalence of pancreatic fistula ranged from 0 to 16%, and the mortality rate varied from 0 to 12.3% in studies with 41 up to 250 patients (Table 2) [7, 14–23]. However, in these studies, the definition of pancreatic fistula was very heterogeneous and mostly not well defined. The prevalence of pancreatic fistula, as determined by the strict criteria of the IGSPF [11], with the modified technique described here, was 8.6% (3 of 35 patients). There occurred two type A and one type B fistula, which all three resolved without any radiological intervention or reoperation. A soft consistency of the pancreatic remnant in ampullary, duodenal, and especially neuroendocrine diseases provide a significantly higher risk of pancreatic fistula [24, 25]. Several groups showed that the rate of pancreatic fistula in high-risk patients with a small fragile soft pancreatic remnant and a non-dilatated pancreatic duct was as high as 36% compared to only 2–4% in low-risk patients with a fibrotic pancreatic remnant composed of dense tissue [26–29]. In the presented series, 10 patients (28.6%) were high-risk patients with soft pancreas texture and only 1 (10%) of them developed a clinically relevant type B pancreatic fistula. However, our technique has to be evaluated in a larger series of patients with this condition to determine its safety.

Until today, there is still no gold standard for the safest technique of PG. The new modified technique described here combines the theoretical advantages of the binding and transfixing modifications, recently reported by Ohigashi et al. and Peng et al. [9, 10]. Only two transfixing mattress sutures are required in our technique, whereas in the techniques of Ohigashi 4 to 6 mattress sutures are placed [9]. Every suture carries the risk for pancreatic laceration resulting in pancreatic leakage, especially in a fragile and soft pancreatic remnant. We also suggest that straight and parallel sutures through the pancreas minimize the trauma to the pancreatic parenchyma and that the gastric wall protects the ligature, similar to felt pledgets in mattress sutures for vascular or heart surgery. The additional purse-string suture in the posterior gastric wall, used in our modification, minimizes any potential space between the gastric wall and the pancreatic remnant. Therefore, two mattress sutures combined with a purse-string suture in the gastric wall eventually prevent damage even to the very fragile parenchyma of a soft pancreas. Our technique provides a theoretical risk of ischemic tissue injury due to the U-sutures. However, we did not yet observe this in our pilot series. Indeed, due to the good perfusion of the stomach wall, the possibility of an ischemic injury at the pancreatic tissue or gastric wall seems rather low to us. Since the transfixing mattress sutures are placed just above the purse-string suture, leakage from the pancreatic stitch holes will drain into the gastric lumen and not outside the anastomosis. The double-armed straight needles, used in our modification, allow an easier handling compared to curved needles. This advantage is clearly apparent, especially when the anastomosis must be performed in unfavourable conditions, such as in the case of an anastomosis of the pancreatic tail into the fundus of the stomach. The placement of a lost drain in the main pancreatic duct will drain the majority of pancreatic fluid in the distal stomach away from the anastomosis, which also might reduce the risk for anastomotic leakage. 

The described new technique is simple and quick to perform. The median time for the PG anastomosis was 18 minutes. This is very similar to the modification of PG described by Ohigashi, who needed 20 minutes for placing four transfixing mattress sutures [9, 30]. The simplicity of our technique might be an important argument for surgical departments with training programs. In case of PJ, Langrehr et al. suggested that the mattress suture technique does not require special training and avoids tangential sheer forces during tightening of the suture thread [30].

One theoretical disadvantage of our modified PG is that a gastrostomy on the anterior gastric wall is required to insure an optimal overview during conduction of this anastomosis. This carries an additional risk for postoperative leakage. However, we did not yet observe a leakage from the anterior gastrostomy. Another potential disadvantage of PG compared to PJ is bleeding from small vessels of the pancreatic stump, which was invaginated into the stomach. We observed this in one (2.9%) of our patients. The bleeding required transfusion of 2 units of red blood cells and could be stopped endoscopically.

## 6. Conclusion

The described modification of PG with two transfixing mattress sutures and one binding purse-string suture at the posterior gastric wall appears to be simple, safe and reliable. Because this paper is preliminary, the presented technique has to be evaluated in larger controlled trials.

## Figures and Tables

**Table 1:** Postoperative complications after the purse-string mattress suture pancreatogastrostomy.

Postoperative complications	Number of patients	%
Mortality	0	0 %
Morbidity	12	34.3%

Postoperative Complications*		

Pancreatic fistulas (PFs)	3	8.6
Grade A	2	5.7
Grade B	1	2.9
Grade C	0	0%
Postoperative Hemorrhage causing Reoperation	1	2.9%
Postoperative bleeding from pancreatic remnant	1	2.9%
Abdominal abscess not caused by PF	1	2.9%
Biliary leak	2	5.7%
Wound infection	2	5.7%
DGE	7	20%

*3 patients with >1 complications.

**Table 2:** Overview on trials on pancreaticogastrostomy.

First author (year)	Number of patients with PG	Number of pancreatic fistulas (%)	Mortality (%)
*Randomized controlled trials*			
Yeo (1995) [4]	73	9 (12.3)	0 (0)
Duffas (2005) [5]	81	13 (16)	10 (12.3)
Bassi (2005) [6]	69	9 (13)	0 (0)
Fernàndez-Cruz (2008) [7]*	53	3 (5.7)	0 (0)

*Observational clinical trials*			
Andivot (1996) [14]	43	6 (14)	2 (4.7)
Kim (1997) [15]	48	1 (2.1)	1 (2.1)
Kapur (1998) [16]	125	0 (0)	6 (4.8)
Fabre (1998) [17]	160	4 (2.5)	5 (3.1)
Arnaud (1999) [18]	80	3 (3.7)	3 (3.7)
Takano (2000) [23]	88	0 (0)	0 (0)
Schlitt (2002) [19]	250	7 (2.8)	11 (4.4)
Aranha (2003) [8]	152	21 (13.8)	0 (0)
Munoz-Bongrand (2004) [20]	242	31 (12.8)	1 (0.4)
Oussoultzoglou (2004) [21]	167	4 (2.3)	5 (2.9)
Hoshal (2004) [22]	84	4 (5)	—
Shinchi (2006) [13]	103	2 (1.9)	0 (0)
Ohigashi (2008) [9]	17	0 (0)	—
Peng (2009) [10]	26	0 (0)	0 (0)
Presented series (2011)*	35	3 (8.6)	0 (0)

*PF classified on ISGPF-criteria.
